# Implementation of a Hybrid Intelligence System Enabling the Effectiveness Assessment of Interaction Channels Use in HMI

**DOI:** 10.3390/s23083826

**Published:** 2023-04-08

**Authors:** Arkadiusz Gardecki, Joanna Rut, Bartlomiej Klin, Michal Podpora, Ryszard Beniak

**Affiliations:** 1 Faculty of Electrical Engineering, Automatic Control and Informatics, Opole University of Technology, 45-758 Opole, Poland; 2 Weegree Sp. z o.o. S.K., 45-018 Opole, Poland; 3 Faculty of Production Engineering and Logistics, Opole University of Technology, 45-272 Opole, Poland

**Keywords:** human–machine interaction, interaction quality sensor, industry 5.0, quality of work, human resource, employee training, co-bots

## Abstract

The article presents a novel idea of Interaction Quality Sensor (IQS), introduced in the complete solution of Hybrid INTelligence (HINT) architecture for intelligent control systems. The proposed system is designed to use and prioritize multiple information channels (speech, images, videos) in order to optimize the information flow efficiency of interaction in HMI systems. The proposed architecture is implemented and validated in a real-world application of training unskilled workers—new employees (with lower competencies and/or a language barrier). With the help of the HINT system, the man–machine communication information channels are deliberately chosen based on IQS readouts to enable an untrained, inexperienced, foreign employee candidate to become a good worker, while not requiring the presence of either an interpreter or an expert during training. The proposed implementation is in line with the labor market trend, which displays significant fluctuations. The HINT system is designed to activate human resources and support organizations/enterprises in the effective assimilation of employees to the tasks performed on the production assembly line. The market need of solving this noticeable problem was caused by a large migration of employees within (and between) enterprises. The research results presented in the work show significant benefits of the methods used, while supporting multilingualism and optimizing the preselection of information channels.

## 1. Introduction

In the developing world of advanced technologies and constantly changing working environment conditions, where time and quality of performed tasks and manufactured products are especially valued, enterprises are forced to search for new means of competitiveness. The advancement is made through the use of new technologies, created for the needs of economic and flexible production while affecting the quality of work performed also by newly hired employees with short work experience. Technical and organizational solutions supported by new technologies created for the implementation of production activities, the application of which is constantly being expanded, are a key element in the development of enterprises that must adapt to the requirements of Industry 4.0 and in the near future also to Industry 5.0.

The ever-changing reality and the evolving digitization of the industry force enterprises to transform toward the inclusion of new and visionary approaches [1,2]. New approaches imply in turn opportunities for enterprises to improve the quality of work organization and/or the selection of techniques that allow maintaining the continuity of the supply chain, and thus, increase the competitiveness of a company [3,4].

One of the foundations of Industry 5.0 is the co-existence and collaboration of men and machines in a natural environment. Controlling and managing the man–machine collaboration process require sufficient methods and ways to be utilized. The authors propose a novel Interaction Quality Sensor (IQS) integrated into the broader system fulfilling the instruction supporting the HMI role in the production line environment. This collaboration is expected to take place using natural communication, instead of specialized interfaces or protocols, shifting the focus towards human-oriented, context-aware, adaptive systems [5]. With the progress of Artificial Intelligence (AI), as well as NLP (Natural Language Processing), which is clearly seen in modern conversational {systems [6,7], the man–machine collaboration becomes an urgent and promising trend in modern industry. Nevertheless, despite recent significant advancements [8,9,10,11] in the field of Human–Computer Interaction (HCI) (namely: the Amazon Echo available since 2015 [12,13,14], Microsoft Cortana massively expanding in 2015 to numerous platforms [15], Google Speech announced in 2016, upgraded in 2018 [16] and 2022 [17], Google Assistant announced in 2016 [18,19], Google Nest introduced as Google Home also in 2016 [20,21,22,23,24], and the Apple Siri updated in 2017 [25,26]) and undoubtful progress in Artificial Intelligence and Machine Learning [27,28,29,30], conversational AI systems usually still tend to disappoint rather than to amaze their interlocutors (and there are some specific reasons for that, already diagnosed and described [31]). In the case of industrial solutions, an appropriate sensor network with insight into every aspect of the man–machine communication with regard to its purpose is required (allowing later to provide insight into the quality and efficiency of every stage of the process for a given worker/trainee). In many cases, the introduction of new tools and technologies into an enterprise ecosystem is associated with the need for a change, including expanding the scope of duties of employees, changing their job position, or opening new job positions focused on knowledge and sustainability [32]. However, intelligent digitization is not about replacing employees with robots or other automatic systems, but about increasing operational efficiency and work comfort by leveraging the importance of flawless and natural man–machine interaction [33]. Appropriately selected tools and IT technology ensure the improvement of the quality of employee workflows, increase the efficiency of the work carried out, improve communication and information flow, and support the development of the enterprise [34,35].

Currently, an increasingly popular way to optimize the work cycle in enterprises and production plants is the use of new technologies and IT tools to support company employees in their daily duties through the use of automation and robotization solutions. Robotics makes it possible to transfer repetitive activities from human employees to robots while allowing to shorten the overall time of process implementation and reducing costs and the number of production defects [36]. Enterprises that want to maintain a good position in the market and gain new contractors must strengthen their image, especially with regard to future prospects [37]. The use of robots increases the production capacity of enterprises, making them attractive partners in a competitive market. The possibilities of using robots in enterprises are even wider, namely, they are currently extremely useful in selected application fields, among others, in quality control processes [38]. The introduction of robots in enterprises contributes to numerous significant benefits, including improved customer service quality, greater motivation of employees to perform their work, as well as a sense of effectively performed work and its optimization [39]. The lifecycle of an enterprise assumes constant evolution toward positive financial income. Nowadays, when the world struggles with pollution and energy price spikes, the life cycle also includes lowering power consumption in the production process. Introducing new technologies such as robotics and AI positively impacts green innovation [40,41].

Despite the popularity of the use of new technologies and the current degree of development of robots, the problem of recruiting qualified employees is still an issue in many enterprises [42]. Many companies and organizations are currently facing difficulties in finding employees with the skills currently in demand [43,44]. Difficulties in acquiring qualified employees may partially be a consequence of the competency mismatch of candidates on the market with the dynamically advancing technological capabilities, digitization, and the evolving specificity and organization of many enterprises [45]. Moreover, the labor market dynamics forces companies to be able to quickly train employees to adapt to production and/or employment changes [46,47], and furthermore, the migration crisis [48,49,50], results in a new requirement—to be able to create workplaces for foreign-speaking employees [51]. All this causes the search for solutions that may support the functioning of enterprises without causing downtime in the execution of tasks. These challenges are met by new technology that offers support and solutions. The current pace of enterprise adoption of new technologies, artificial intelligence, robotics, and automation in the workplace is strongly accelerating [52]. The whole range of opportunities related to new technologies is changing. Artificial Intelligence, as well as robotics, open up new opportunities for many areas of business operations [53], including the HR department [54,55,56,57].

Mitigating human resource shortages requires the entrepreneur to make difficult decisions about delegating qualified employees to train new workers, whose integration with the workplace environment and processes can itself become a long-term task, which carries the risk of failure [58]. The training abilities of the senior staff directly affect not only the success rate achieved, but also the newly established relationships with new employees, and the level of adaptation of training to the skills and knowledge of a particular person [59,60]. The number of quantified parameters affecting successful training in the above case increases with the complexity of the activities performed at the workplace and the possible level of knowledge absorption by a person per unit of time. The unavailability of communication in the employee’s native language (and insufficient adjustment of training methods and materials) is a serious barrier that is difficult to eliminate [61].

In the case of a classic approach (employee training without involving advanced IT systems) to the management of the employee training process, the evaluation (assessing the effectiveness of the teaching-learning process) and the prediction of their achieved proficiency level are difficult to implement (mostly due to the lack of appropriate procedures implementations, as well as reliable data) [62]. Nevertheless, new technologies based on Machine Learning [57], predictive analytics [63], and Big Data [56] can significantly improve employee onboarding processes within an organization [64,65]. Moreover, they can also provide insight into the KPIs (Key Performance Indicators) of the implementation process [66], which allows for systematic standardization of methods and teaching materials used in training [67], and for adapting the information transfer process to the individual predispositions of a particular individual (e.g., predominance of visual- or speech-based form of information, the speed of information transfer, etc.) [68]. An additional noteworthy aspect in the field of production engineering is the possibility of providing a training stand able to take its parameters into account at the stage of simulating the influence of the new employee on the production line operation. This will be possible with the help of the Interaction Quality Sensor (IQS) proposed in this article, which introduces the implementation of Hybrid INTelligence (HINT) architecture for intelligent control systems, enclosing the prioritization of information channels and optimizing both the apprenticeship of unskilled workers and the performance of employees.

Industry 5.0 builds on the previous industrial revolutions but focuses on integrating new digital technologies to create an intelligent and flexible production system. Industry 5.0 aims to create an intelligent factory that is fully integrated and allows employees, machines, and IT systems to collaborate in a more harmonious way. This integration can lead to improved efficiency, reduced waste, and increased sustainability. The presented HINT system follows this trend by implementing the proposed adaptive mechanism in the area of information channels used in HMI. The HINT system uses multimedia information (e.g., speech, images, videos), as well as optional co-bot support, especially for applications with foreign and/or unqualified employees.

## 2. Materials and Methods

Production quality, often depicted by the technical quality of a product, seems to be quite an intuitive term. However, the evaluation of employee training quality is not trivial [69], and it may be not suitable for intelligent control of the training process unless it is properly approached, defined, and measured. This ‘training quality’ can be perceived as a result of (1) the training/teaching process (initial basic training as well as the mastering performed later) and (2) the personal predispositions of a particular employee, play an essential role in the decision-making process of the HINT system algorithm relaying especially on sensing capabilities of the IQS. The production line‘s sensors provide the knowledge of the apprentice’s work quality [64] and enable adjusting the steering parameters for HMI guidance delivery channels, conceivably due to the HINT system’s capability of full integration into the factory processing event bus. Based on the available information, the HINT system is designed to be able to distinguish alternate information channel scenarios and to utilize a control algorithm optimized with regard to a particular apprentice and the situation. The IQS delivers the necessary data by comparing patterns of behavior saved in the set of basic instructions against the behavior of the worker and expected quality in advancements of current activity. The optimization process of selection of the best-performing channel to the situation conditions was already briefly introduced in [64], and presented by comparing it to the concept of Hanoi Towers. Meanwhile, the study presented in this article refers to the real-world conditions of a local furniture factory and the conclusions drawn from real-world use-case implementation and integration with an existing quality control system, by enhancing the functionality of an existing apprentice workstation with HMI instruments, and upgrading it to a quality- and process-related research station. The factory was categorized as having: (a) medium complexity of operation producing wooden windows and doors for civil houses—manually assembled using prefabricated parts and specialized tools, (b) a significant level of job quitting individuals just after initial training, and (c) a long (unacceptable) time of training before reaching the required level of self-sufficient proficiency at work.

**Figure 1:**
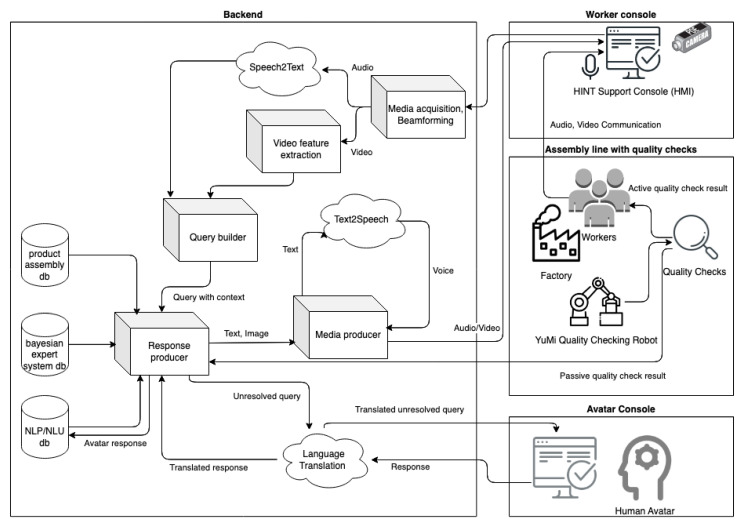
Main components diagram of the Hybrid Intelligence System (HINT).

An important part of the experiment was the customized HINT system software, prepared and installed in the HMI workstation (see Section 2.4), providing work guidance/instructions to novices in textual, vocal, and visual form (both static and moving images)—in frames of a tutorial, especially considering preferences and the predispositions of the individuals, while recording their choices and performance (language, menu navigation, information channels choices, perception delay [70], assembly (execution) time, etc.). In an emergency (when no further steps of assembly could be reached), there was a possibility of a two-way voice connection to the foreperson for instructions/support. Communication between HMI and workers could be done by voice commands or touch screen. The transcripts of all interaction events were visible to the manager (foreperson) in a web browser window, along with the camera top view of the workstation, and the current state of all workstation screens. The foreperson was able to interfere with the communication of the system and the worker, by using a web chat window, to make adjustments (or even overtake) the system’s guiding role for a while. This functionality was also designed to introduce amendments that could actively retrain the underlying neural network model of the expert system (Figure 1). During the experiment run, it was recorded which elements of the system were used by the apprentice, for how long, and how many times (the most important aspect of the data collected was the use of information channels). The acquired performance data were linked to the result of the final assembly quality check, which allowed conclusions to be drawn. In pursuit of remarkable level of User eXperience of man–machine communication, the authors proposed a solution incorporating the following ideas [64]:

- AI system, with a predefined fact database containing topics within an expert knowledge domain;
- The ability to extend/supplement the system’s knowledge by adding new facts and relations;
- A human-in-loop expert assistance for the approval of new knowledge and for emergency situations.

### 2.1. Hybrid Intelligence System (HINT) Architecture

The concept described in the current research aims to enable the adaptation of a system to users’ preferences and to improve the operation of the system as a result of the analysis of the process data. The system analyzes the initial stages of interaction and the user’s preferences (choice tendencies) and adjusts the presentation of content (resources) according to a personalized interaction model. The patterns are based on the data from previous interaction instances.

The developed HINT system allows for a variety of architecture configurations corresponding to the characteristics of the implementation requirements in a given production environment. As part of the system validation, the HINT system was implemented in the woodworking industry, specifically in a company producing wooden joinery. The specificity of the company focused on the implementation of short series and products, and even the production of personalized products, is a good training ground for the developed system.

The implementation of the HINT system, which is especially important in the face of the new multilingual nature of the labor market, takes the full benefits of the modular system architecture (presented as a simplified diagram in Figure 1). The following modules are involved in the personalization of user interaction: STT, TTS, data repositories, quality assurance module, and others.

The HINT system has been designed to be modular, allowing it to be adapted to customer requirements. One of the modules is responsible for system integration with external devices and systems. As an example of its versatility and modularity, the ABB YuMi co-bot can be used, which was successfully applied in data acquisition for active verification of assembly quality (as presented in Section 2.4).

### 2.2. Dual Sensor Component: Interaction Quality Sensor Merged with User Experience Sensor

**Figure 2:**
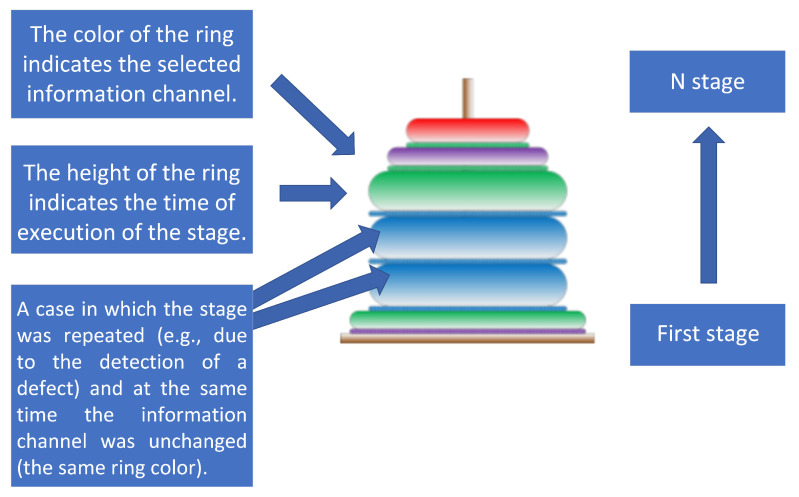
Graphic explanation of the idea of reflecting the process as an analogy to the towers of Hanoi presented in [64].

The presented implementation uses the algorithm described in [64], designed as a dual mode sensor including User Experience (UX) sensor and Interaction Quality Sensor (IQS). The idea and theoretical basis of this sensor are described as an analogy to the towers of Hanoi (see Figure 2).

This is initiated automatically without the knowledge and awareness of the system user—which is fulfilling the role of the User Experience Sensor.

The primary objective is to reconsider the adaptation of the information channels to best-performing in the context of individual preferences of the user on the basis of the readouts of IQS/UX sensors. At a glance, the best-performing channel is selected after analysis of the retrospective data of previous stages of completed work as presented in Figure 2 using the Hanoi Towers approach. Exemplary adaptation of HMI guidance delivery channels in case of lack of progress (no switching to the next stage) in a given assignment would proceed as follows (described in detail later in Section 2.4):

1. If a trainee is stuck in a given stage and decides to try a different information channel, which is visualized in the Hanoi Tower as the same width of disks (the tower does not narrow up, the subsequent disk is of the same width, which represents a repetition of the stage);
2. The work performance at a given stage performed by a given trainee is acquired by the proposed IQS and these data increase the knowledge base about the trainees’ performance per stage;
3. The best-performing channel is considered for pre-selection as the default one for future users.

The secondary objective is to detect probable faults in users’ work as quickly as possible by comparing the path and timing of activated sections on the video stream between recorded traces of workers and saved patterns within the instruction. The found discrepancy is marked for verification and inspection in case of any automated quality checking system within the production line. Later, aggregated data of discrepancy of a larger count of complete users’ interactions are analyzed in terms of the quality of the interaction. High values would mean that instruction is not explicit or does not fit the personal capabilities of an individual.

In addition, data from this process can be used to optimize the process according to selected criteria, e.g., time, process quality, or both of these factors at the same time. Testing the performance of this sensor in optimization issues will be the subject of future research.

The process of building the tower of Hanoi can be used for representing any process implemented in the system. For proper analysis of interaction processes depicted using Hanoi towers (as in Figure 2), the following assumptions should be made:

1. The diameters of the rings placed on the tower are adjusted to the number of stages in the analyzed process;
2. The largest diameter ring corresponds to the first stage of the process, with rings gradually decreasing in size with each subsequent stage;
3. The height of each ring represents the duration of execution of each stage of the process;
4. The colors of the rings represent the particular chosen information channel, such as text, images, videos, or consultation with an expert.

### 2.3. Vision System as an Additional Detector of Process Anomalies

The IQS proposed in this article can optionally be equipped with a visual analysis subsystem of the correctness of assembly process stages, in order to facilitate, streamline, and automate the verification of the technical correctness of a given process stage. The inclusion of a video supervision subsystem significantly supports the operation of IQS, offering additional insight into the quality of work (or employee training).

### 2.4. Adaptive Information Channel Customization Module Based on the “Hanoi Towers” Concept

**Figure 3:**
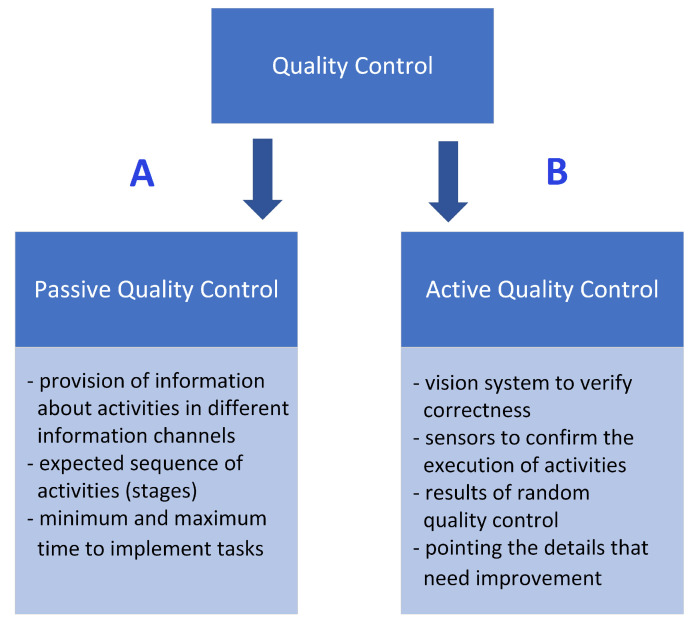
(**A**) Passive and (**B**) Active Quality Control in the exemplary implementation of the HINT system in a wooden joinery production industry company.

Quality control may be defined as a process that helps a company make sure that it creates quality products and that its employees make minimal mistakes. Therefore, in order to achieve this goal, two mechanisms have been distinguished: Passive Quality Control (Figure 3) (mechanisms supporting the employee by providing them with the necessary information, forcing the correct order of operations, etc.) and Active Quality Control (understood as active control and verification). The proposed implementation uses a vision system that analyzes the correct assembly of the key node.

During the research and development of the HINT (Hybrid INTeligence) system, (created as part of research at Weegree company, and implemented, tested, and validated at Halupczok company), numerous solutions were designed and applied in order to support the new employees during the process of getting acquainted with the new workplace and to support the quality control during the training and the performance of work. The HINT system is capable of using a co-bot to support active quality control (Figure 3B) by using vision processing. Passive quality control (Figure 3A) is carried out by enforcing the correct order of activities and is supervised by the HINT system.

**Figure 4:**
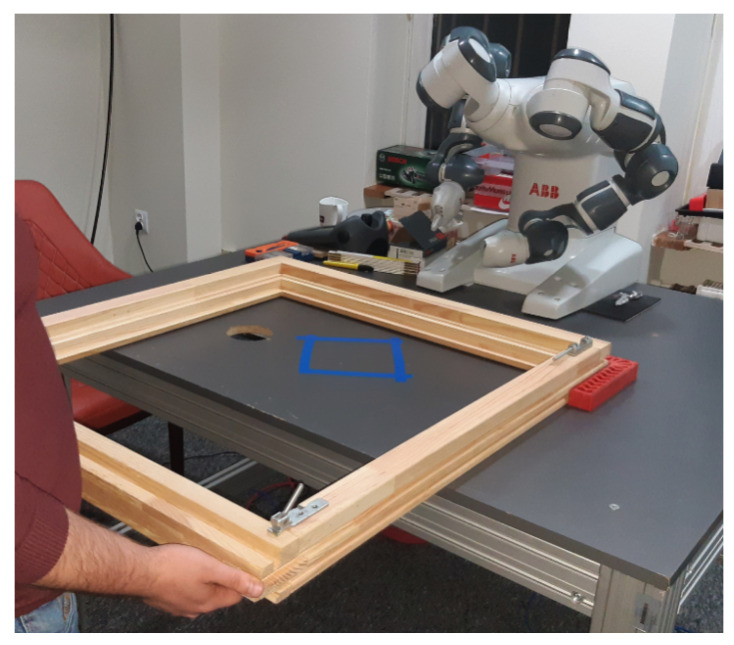
The co-bot interactive helper workbench. The interaction interface hardware is the same as presented in the next figure.

The quality assurance module uses two mechanisms implemented in the HINT system: (1) passive quality control, which is carried out by enforcing the correct order of activities (stages) (supervised by the HINT system), and (2) active quality control (presented in Figure 3) using video analysis. In one of the test implementations of the system in the woodworking industry, a vision sensor integrated into a SmartGripper attached to the ABB YuMi co-bot (Figure 4) was used. It acquires visual data for image processing enabling the assessment of the correctness of the key stages of assembly of the wooden window sashes and frame. The YuMi co-bot operates in accordance with the guidelines of the ISO 10218-1 standard [71] for safety of people in a previously defined (at the stage of designing the workplace) workspace [72].

The HINT system, in the case of an employee’s need to contact a supervisor (or other competent person regarding the performed tasks), provides the possibility of a remote teleconference connection with an expert. This mechanism supports and implements the above-mentioned active quality control. Employees were able to contact an expert (called Avatar) when they encountered a difficult problem. The assembly training was monitored independently. During the initial phase of testing, before optimization, employees were given the autonomy to decide when to utilize the Avatar assistance. However, after the optimization, the HINT system was able to identify the Avatar to be the most appropriate information source and prioritized it higher than other information channels.

**Figure 5:**
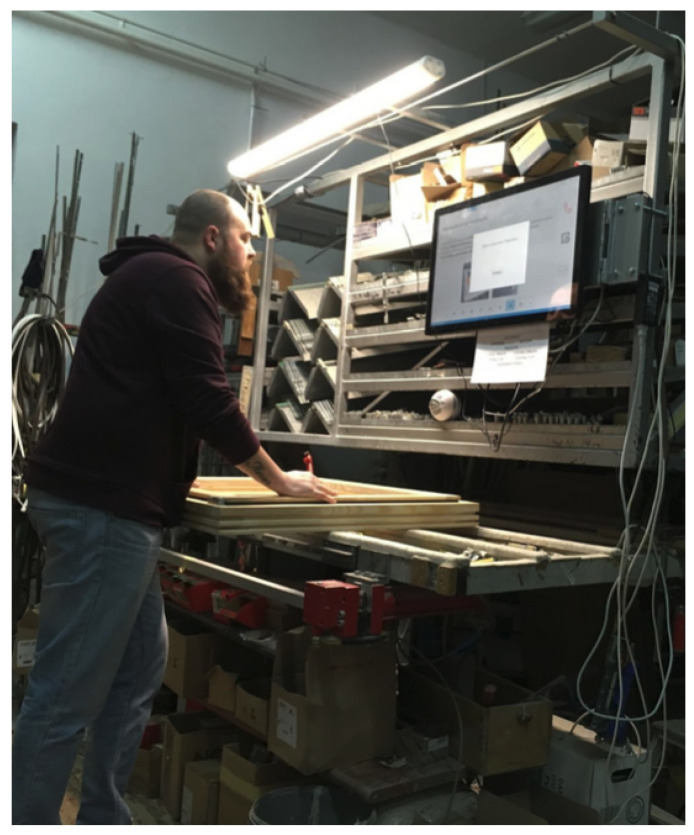
Interactive assisted assembly stand.

**Figure 6:**
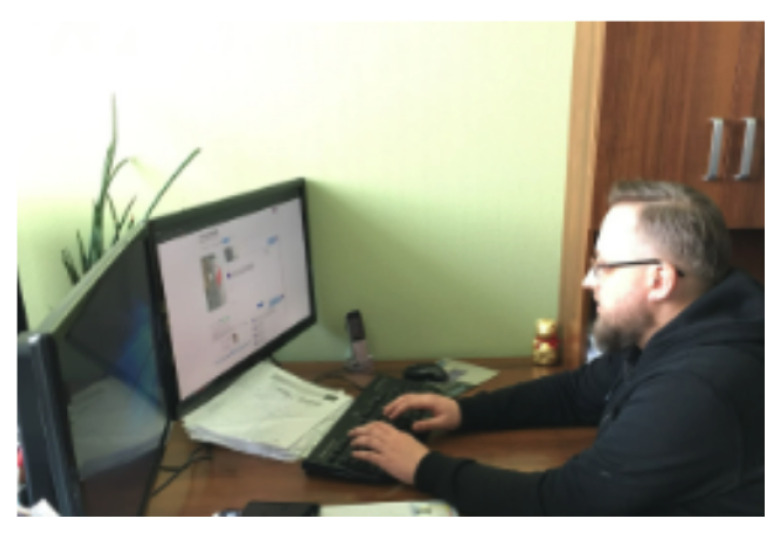
The employee’s last resort—a videocall with the expert (the expert’s workplace and communication application interface).

The analyzed experimental interactive assisted window assembly process consisted of twenty separate assembly steps, and was carried out at the Halupczok company production facility. A number of inexperienced people (with no prior experience in window assembly) faced the challenge of completing the task with the help of the interactive stand (presented in Figure 5). They were briefly instructed on its HMI interface, available information channels, including the last option (which would on the one hand make the assembly process possibly take longer, but on the other hand could be helpful in avoiding mistakes or damage)—the Avatar support (Figure 6).

### 2.5. User Interaction Adaptive Model Implementation

The Hybrid Intelligence System (HINT) was first introduced theoretically in [64], where the authors presented a novel user experience optimization concept and method, named the User Experience Sensor. The concept presented there enabled the selection of the most effective channels of information transfer [73] available to be chosen from for any given stage:

- Text-based information (description);
- Speech (TTS synthesized speech audio);
- Images (visual guide with photos or diagrams);
- Videos (mostly available only for stages that are difficult to explain);
- Video call to the senior staff or process supervisor (VOIP audio connection with the additional top-view camera of the workbench).

Choosing and switching between available information channels (forms of information for every given stage of window assembly) helps to optimize the efficiency of a production line (for a given employee) or of training of a particular new employee.

In the implementation of the HINT system, the concept of quick employee onboarding—regardless of their current technical competencies and regardless of what languages they speak—was an absolute priority. The specificity of the local labor market consists of the availability of workforce; however, with limited knowledge of the local language (in this case, Polish). The system has a module that allows you to quickly change the predefined languages (languages of text messages, verbal messages, visual content). Verbal communication with the system also requires access to the STT service in the selected language.

An important element of the employee support system (Figure 5), especially at the initial stage, is the ability to contact an expert directly using the interactive workbench (Figure 6), and while multilingualism is supported, it is possible to translate the user’s and/or Avatar’s statements on the fly in the event of a language barrier using STT and translation and TTS. A translation module based on the Google Cloud service is used for this.

**Figure 7:**
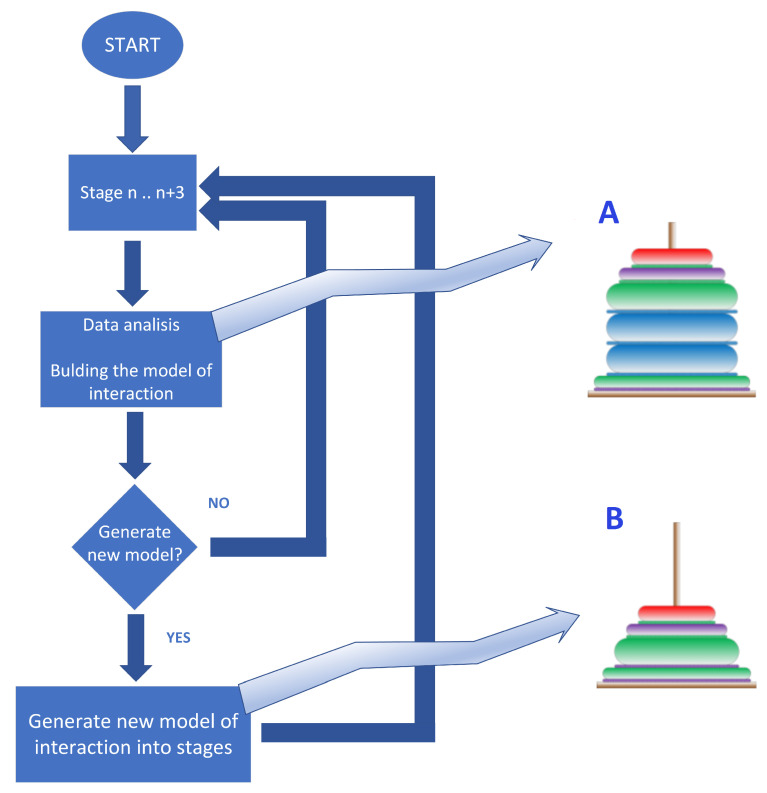
The adaptive matching mechanism of the presented content to the detected user interaction model: (**A**) before optimizations, (**B**) after optimizations.

At the beginning of every training (the system operation), the personal preferences of individual employees are unknown. This is a result of individual cognitive preferences and so-called Default Bias [74,75] in the user expectancies towards interactive systems or kiosks. Therefore, the system starts working on the default configuration of information channels used to present content (Figure 7A). As a result of the analysis of data from the first stages (e.g., typically the first three stages, but the range can be adjusted), the system compares the particular employee’s interaction model and, if it deviates from the proposed model, reconfigures the information channels to adapt to the employee’s individual preferences (Figure 7B).

This tactic can be used many times during the user’s activity after collecting and analyzing each portion of information about the preferred information channels used. This mechanism also modifies the statistical model of the default configuration of information channels, which can be modified for use with selected cohorts (e.g., age- or language-related groups). The “default configuration of information channels” is understood as a predefined information channels-related communication scheme consisting of available channels (audio, video, graphics, and selected language) prioritized in a predicted/presumed order. When the system determines that the window fittings have been properly installed, it selects as default those forms of providing information that reduced the work time of the trained person while correctly completing the assembly stage [64]. The system was prepared to operate in four predefined languages or in the case of calling an avatar that does not speak the language of the learner, we have a multi-step process consisting of: STT—Google Translator—Avatar answer—STT—Google Translator—TTS. The adaptive mechanism of the method of choosing appropriate information channels supports the minimization of the negative effects of the Default Bias, which consists of imposing on the user an inadequate (not optimal) information channel choice, resulting from the statistical analysis of the generalized behavior of cohorts (Figure 7).

## 3. Results

The HINT system, presented in Section 2 (and its architecture—in Section 2.1), was included in the design of the prototype of the Adaptive information channel customization module (Section 2.4), which enabled the possibility to perform experimental research on the User interaction adaptive model (Section 2.5). The results of the trials—using HINT to leverage the efficiency and quality of training of new (unqualified) employees for a particular task—are presented below.

### 3.1. Use Case 1—HINT as a Window Assembly Assistant

The first use case process—onboarding of untrained employees using the HINT-equipped windows assembly interactive assistant—was conducted twice: once before optimization and once after, and the employees were not trained beforehand. The progression of each stage was recorded. Additionally, each user was asked to fill out a process evaluation questionnaire, which served as the foundation for the User eXperience (UX) evaluation. After finishing each HINT-assisted window assembly training session, the quality of each window assembly was assessed.

The experiment participants were chosen at random from a group of volunteers with no prior experience in window assembly. They were only given the HINT system, available on a touch screen with textual instructions and images for each assembly stage, and videos available for the majority of steps. Each employee was instructed how to interact through voice commands and the touch screen.

### 3.2. Use Case 2—HINT with a Window Assembly Co-Bot

The proposed HINT system was also tested with the use of ABB YuMi co-bot (see Figure 4) to investigate its purposefulness in operation with co-bots (cooperative robots). The YuMi co-bot was intended to help the human employee in the window assembly learning process, by participating in the assembly. However, it turned out that the inclusion of a robotic companion adds some complexity to the learning process. The basic part of the research (analysis, comparison, and optimization of the information channels in the training process) was designed and conducted without a co-bot and contributed the most interesting and valuable insights into HINT-assisted employee training. The inclusion of a co-bot introduces new interactive training options, extending the set of available information channels.

### 3.3. Analysis of the Learning Process Using HINT

The impact of the learning process using the HINT system depends on the participants’ perception of the difficulties of the technological process as well as the quality of the documentation prepared by a foreman (supervisor of the teaching procedure and expert) for mastering that process. For the window assembly process, which was used as the research use case basis for the analysis of the HINT system operation, the trainees/participants stated that the performed activities were difficult or very difficult, and the quality of the prepared documentation was good or very good. This allows for the conclusion that the perception of the quality of the instructions translates into actual actions resulting in the quality of window installation. This subjective assessment was confirmed during the real-life experimental verification. The correlation coefficient between the perceived quality of the instruction and the verified quality of the assembly was 0.69—both for the assemblies performed without the HINT system (only with paper documentation: text, illustrative photos, or in-person dialogue with an expert), as well as in the case of its use within the training process (information channels: text, illustrative photos, voice messages, illustrative video, or remote video conference with an expert).

**Figure 8:**
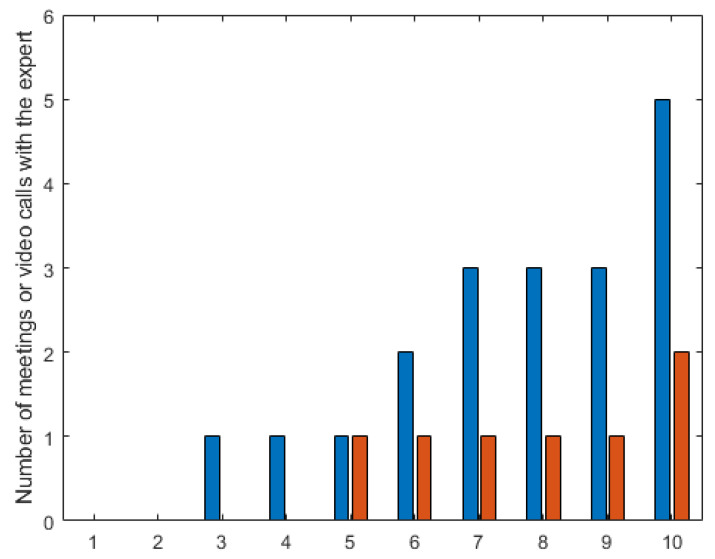
Number of meetings or video calls with the expert for individual participants. Blue bars—without the use of the HINT system, only paper documentation (information channels: text, overview photos) or physical contact with an expert. Red bars—with the use of the HINT system (information channels: text, overview photos, voice messages, overview video, remote contact with an expert). Data arranged in ascending order.

Important information is that in the case of working with the HINT system, the ability to adjust information channels resulted in a significant decrease in the need to use time-consuming contact with an expert. The average time of a single contact with an expert decreased from about 62 s if the HINT system was not used and to 26 s if this system was used (Figure 8).

**Figure 9:**
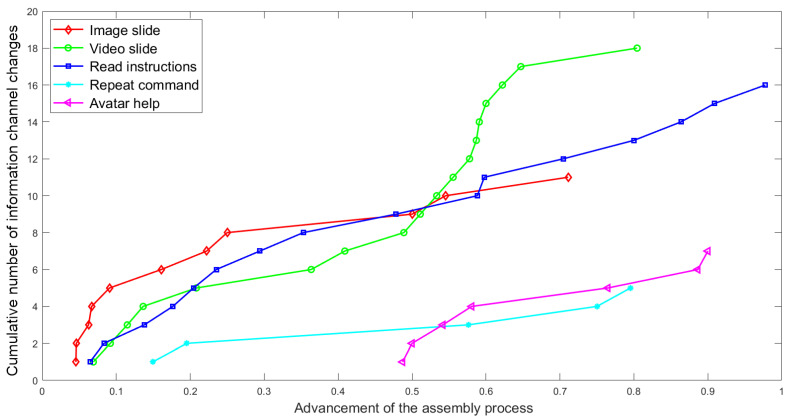
The number of changes of information channels depending on the advancement of the window assembly process using the HINT system).

In the case of using the HINT system, an interesting information source usage specificity on window assembly can be observed. This specificity is illustrated by the Figure 9, which in a synthetic way shows the use of the information channels by all participants/trainees as the window assembly process progresses. Such determination of the independent variable results from the fact that there are significant discrepancies in the interaction with the HINT system by various persons. The smallest number of interactions was 10, while the largest number was 92.

Figure 9 shows that interactions involving the use of the vocalized description of an assembly step (blue) occur uniformly throughout the assembly process. At the same time, as the window assembly process progresses, the use of videos (green) increases (the number of uses is numerically highest at the end of assembly). In the initial stages of assembly, pictures and diagrams constitute a vast majority of information channel choices, which decreases (at about 2nd/3rd of the assembly process), along with the “discovery” of other, in the opinion of the learners, more advantageous forms of transferring information. The figure also shows that the repetition of the information transfer process (cyan) usually occurs in the middle of the assembly process and is not used at the beginning when the assembly steps depicted seem to be easy for the assembler. This may also be caused by fatigue of using one information channel, mitigated by using another one; however, the authors tend to see this situation rather as a search for better explanation of assembly steps. Asking for help from the supervisor (magenta) starts to appear about halfway through the process and initially aligns with the repetition of information delivery and then supplants the repetition process as some trainees have found this assistance more useful than repetition of the information delivery process in any other form. Table 1 contains detailed information on the number of uses of various forms (channels) of information retrieval by 10 selected unqualified participants of the window assembly experiment involving the use of the HINT system.

Table 1 shows that there were people who often used different information channels, e.g., participants 1 and 4. There were also people who preferred one specific form (channel) of information, for example, participant 6 and 9.

## 4. Discussion

The purpose of this paper was to present an implementation of Hybrid INTelligence architecture for Intelligent control systems (HINT), enclosing the prioritization of information channels, and optimizing both the apprenticeship of unskilled workers and the performance of employees.

The HINT system presented in the article and its exemplary implementation in the production environment is an example of a solution that naturally fits into the industry 4.0 concept. The modularity of the system facilitates its adaptation to particular use-case-specific implementation requirements, and at the same time enables the system to be extended with new functionalities. An example is the adaptive content customization module based on the concept of “Towers of Hanoi” (presented in [64]). Integration of applications originating from diverse implementation areas into common quality-oriented training systems (similar to the HINT presented in this study) seems to be the transcendent direction in which many other employee-supporting systems will follow.

They contribute to an increase in employee efficiency, ensure the quality of performed tasks and enable the labor market to absorb new employees (despite their initial lower competencies and/or a language barrier).

Looking into the future of development of processes containing a human element and trying to visualize the latest human-centric technological trends, the proprietary HINT system presented in this study offers the opportunity to improve the quality of training, as well as the effectiveness of human work and enables easier adaptation of a person in the process of performing the required activities by selecting the most effective information channels and methods of knowledge transfer, carried out without the participation of experts.

The HINT system was developed with thought and intention to leverage the teaching effectiveness of multimedia information channels, such as speech, images, and videos (and optionally the assistance of a co-bot), particularly for applications with inexperienced employees (with limited skills or with language barriers). HINT aims to enable employees and aid enterprises in efficiently integrating new hires into the works and processes performed.

The proposed solution in the form of the HINT system is part of the future development of robotization, automation, and the upcoming Industry 5.0. The HINT system has many possibilities that enable the quick assimilation of information required in the implementation of tasks in places where employees are required for various reasons, including economic ones. Current studies of the use of the HINT system indicate an increase in the speed and efficiency of knowledge acquisition.

Industry 5.0 gives new opportunities to obtain high added value of human capital as an element that gives value to manufactured products [76]. The idea of Industry 5.0 emphasizes the need for cooperation between man and machine, while pointing to the importance of humans in the manufacturing processes. The idea of people and machines working together leads to flexible and effective business models [77,78,79,80,81,82,83].

### 4.1. HINT’s Placement in the Kaleidoscope of Robotics’ Future

Currently, enterprises operate in times of rapid technological progress, in particular the development of digitization driven by the need to reduce energy consumption, maximize efficiency, and react faster to changes in the immediate and global environment. The events of 2021–2022 significantly changed the list of issues to be considered when planning business continuity, i.e., dynamic adaptation to new conditions and ensuring sustainable development.

Ideas based on automation, robotization, and artificial intelligence are already used in the Industry 4.0 trend and will, thus, dominate the upcoming industry of the future. Currently, the possibilities of new technologies and the technological potential of robots have reached an unbelievable level of development, and their general availability in connection with large adoption (almost indispensable) in enterprises has a critical impact on the development of many industries. Robotics, automation, and AI are already changing the quality of life in a wide range of activities [84] and the way work is performed, increasing the level of efficiency and safety of activities and processes, improving the standard of services, customer service, and logistics, and improving the supply chain.

Cooperative teams, consisting of humans and machines (robots), can be seen to constitute interconnected cognitive entities, able to include human communication using natural language. With the communication performed purely using voice or text communication, as well as answering questions and giving recommendations for action, cooperative systems are able to use their knowledge and capabilities to contribute to increasing the quality of manufactured products, optimizing the implementation of activities and making better decisions (both strategic and tactical) by the management staff [85]. The progress of digitization, new technologies within the Industry 4.0 and the idea of Industry 5.0, automation, robotization, and use of AI require the use of the Internet, which results in the dawn of a new era of human–robot interaction, IoT, security, cybersecurity, work performance, functioning, and operation.

In the last few years, new types of interactions between humans and machine learning algorithms have emerged, which can be grouped under the umbrella term human-in-the-loop (HITL) [86,87]. HITL is a branch of artificial intelligence that requires the involvement of both humans and machines to create machine learning models [88]. HITL illustrates a process where a machine or computer system is unable to solve a problem on its own and requires human intervention. The human, then, becomes the element that trains, adapts, tests, and in many cases also controls the system’s algorithms. The HITL process is a continuous feedback loop, which means that every training-and-test task is fed back into the algorithm. HITL aims to achieve what neither man nor machine can achieve in isolation, i.e., by functioning independently. An effective HITL system is designed in such a way as to allow many people to contribute information to the learning process (at any time), and the person giving feedback is usually responsible for making the final decisions about the learning process [76,89,90]. By applying the HITL concept, HINT becomes more capable of performing a variety of tasks by facilitating employees’ cognitive (including knowledge) and physical (mainly manual and motor) skills, while profiting from the robots’ ability to perform repetitive tasks and tedious activities [91]. The advantage of using HINT is the ability to parameterize the quality of the job instructions provided, and more precisely to identify elements that need improvement because they are incomprehensible or complicated for the employee, which is visible through the excessive use of communication channels around one stage. If the obtained information is used to update work instructions, the instruction standardization process can be carried out. The ubiquitous profiling (it is common in the world of the Internet to learn the preferences of the Internet user for marketing purposes) is also reflected in HINT, but in a fruitful way for everyone—allowing for the personalization of the process of transferring knowledge to the user, enabling them to learn quickly, and hypothetically reducing their stress and tension. HINT is a system that connects a cooperating man with a machine into a team and provides the function of a supervisor, thus relieving the qualified management staff.

### 4.2. Safety of the System (Human and Robots Cooperation)

Measures to ensure safety during human–machine cooperation are described in the ISO 10218 standard [71,92] (entitled “Robots and robotic devices—Safety requirements for industrial robots”). This is an international standard for the safety of industrial robots, developed by ISO/TC 184/SC 2 “Robots and robotic devices” in parallel with the European Committee for Standardization. It consists of two parts: (1) “Robots”, describing the requirements for robots, and (2) “Robotic system and integration”. Together, these standards contain requirements for robotic systems, including systems cooperating with robots. The standard is aimed at robot manufacturers, integrators, and builders of workstations containing robots and co-bots. According to the [93], a collaborative robot workstation “should be considered as a machine or assembly of machines, depending on the degree of complexity”, which implies the necessity to extend the scope of considerations—from “a robot” to “the system”.

### 4.3. The Co-Bots’ Future and the Road Map to Industry 5.0

Presently, a crucial aspect in the advancement of businesses is the integration of machines, robotic systems, people, and processes [94,95,96], resulting in an increased utilization of co-bots. The incorporation of robots and co-bots in businesses brings numerous benefits for both the enterprises and their employees. In addition, the lack of qualified employees means that companies increasingly decide to automate processes, including the use of robots, which begin to play the role of collaborators, with their potential recognized by entrepreneurs. Human–robot collaboration sets a new direction of activities in which human–robot cooperation takes place [91]. Collaborative robots (co-bots) are machines with technical precision in performing tasks, designed to work safely with people in human-centric environments [97,98,99,100,101,102]. Co-bots, due to their flexibility, neatness, and light mechanical structure (low weight, small size, ease of use, and being extremely precise) can be introduced in any enterprise and used for various processes and tasks, practically at any time [103,104,105,106].

## 5. Conclusions

This article is the summary and the result of a new approach to the optimization of man–machine interaction in collaborative, Industry 5.0-compliant workplaces. The authors propose not only sensing the quality of individual tasks as “building blocks” of the process’ representation, but also adding a supervising layer able to evaluate, manage, and optimize the execution of individual blocks (stages) as well as the whole interaction.

One of the results of the research is the ability to model the window assembly process (or any given work task) using the proposed quality parameters, visualized as the “building blocks” of a specific height and width.

The most important research result (and the research contribution) is the ability to use the proposed process model to automate the optimization, taking into consideration the individual preferences of particular participants, easily introducing human-centricity and collaborative intelligence into the Industry 5.0 workflow.

## 6. Future Work

The main challenge for upcoming research will be the adaptation and profiling of the proposed architecture to specific use cases of new workplaces. Further improvements and advancements will include, inter alia: centralized AI engine for numerous domain-restricted agents [107], distributed implementation for the centralized AI engine [108], load optimization for the distributed engine [109,110,111,112], integration of interactive artificial agents (including co-bots), and possibly a better data representation model created using information retrieval logic and a specified description language [113,114] instead of a static database of facts and relations. An alternative approach would be to follow the mainstream of current AI research and to implement a GPT-3-based chatbot [115]; however, this approach either needs reasonable funding or will generate a general model not suitable for domain-specific professional uses. Therefore, the future of assisted quality-oriented employee training systems remains an open topic.

## Figures and Tables

**Table 1:** Summary of the number of the information channel changes by the assembler.

Participant	Images (Graphical) [No. of Uses]	Videos (Multimedia) [No. of Uses]	Audio (Speech) [No. of Uses]	Channel Replay [No. of Repetitions]	Avatar Teleconference [No. of Uses]
1	4	4	4	2	1
2	0	0	1	0	2
3	0	0	1	0	1
4	2	3	2	2	0
5	0	0	0	0	1
6	0	1	5	0	1
7	0	0	0	0	1
8	0	2	1	1	0
9	3	7	1	0	0
10	2	1	1	0	0

## Data Availability

Datasets and source code are unavailable due to intellectual property.
